# Strategies to Integrate Genomic Medicine into Clinical Care: Evidence from the IGNITE Network

**DOI:** 10.3390/jpm11070647

**Published:** 2021-07-08

**Authors:** Nina R. Sperber, Olivia M. Dong, Megan C. Roberts, Paul Dexter, Amanda R. Elsey, Geoffrey S. Ginsburg, Carol R. Horowitz, Julie A. Johnson, Kenneth D. Levy, Henry Ong, Josh F. Peterson, Toni I. Pollin, Tejinder Rakhra-Burris, Michelle A. Ramos, Todd Skaar, Lori A. Orlando

**Affiliations:** 1 Duke Department of Population Health Sciences, Duke University School of Medicine, Durham, NC 27701, USA; 2 Durham VA Health Care System, Durham, NC 27705, USA; 3 Center for Applied Genomics & Precision Medicine, Duke University School of Medicine, Durham, NC 27708, USA; olivia.dong@duke.edu (O.M.D.); geoffrey.ginsburg@duke.edu (G.S.G.); teji.rb@duke.edu (T.R.-B.); orlan002@duke.edu (L.A.O.); 4 Division of Pharmaceutical Outcomes and Policy, Eshelman School of Pharmacy, University of North Carolina at Chapel Hill, Chapel Hill, NC 27599, USA; megan.roberts@unc.edu; 5 Regenstrief Institute, Indianapolis, Indiana University School of Medicine and Clem McDonald Center for Biomedical Informatics, Indianapolis, IN 46202, USA; prdexter@regenstrief.org; 6 Center for Pharmacogenomics and Precision Medicine, Department of Pharmacotherapy and Translational Research, University of Florida, Gainesville, FL 32610, USA; aelsey@cop.ufl.edu (A.R.E.); julie.johnson@ufl.edu (J.A.J.); 7 Institute for Health Equity Research, Icahn School of Medicine at Mount Sinai, New York, NY 10029, USA; carol.horowitz@mountsinai.org; 8 Division of Clinical Pharmacology, Department of Medicine, Indiana University School of Medicine, 950 W. Walnut Street, Indianapolis, IN 46202, USA; kenl330@yahoo.com (K.D.L.); tskaar@iu.edu (T.S.); 9 Department of Biomedical Informatics, Vanderbilt University Medical Center, Nashville, TN 37232, USA; henry.h.ong@vumc.org (H.O.); josh.peterson@vumc.org (J.F.P.); 10 Division of Endocrinology, Diabetes and Nutrition, Department of Medicine, University of Maryland School of Medicine, Baltimore, MD 21201, USA; tpollin@som.umaryland.edu; 11 Department of Population Health Science and Policy, Icahn School of Medicine at Mount Sinai, New York, NY 10029, USA; michelle.ramos@mountsinai.org

**Keywords:** genomic medicine, clinical decision support, implementation science

## Abstract

The complexity of genomic medicine can be streamlined by implementing some form of clinical decision support (CDS) to guide clinicians in how to use and interpret personalized data; however, it is not yet clear which strategies are best suited for this purpose. In this study, we used implementation science to identify common strategies for applying provider-based CDS interventions across six genomic medicine clinical research projects funded by an NIH consortium. Each project’s strategies were elicited via a structured survey derived from a typology of implementation strategies, the Expert Recommendations for Implementing Change (ERIC), and follow-up interviews guided by both implementation strategy reporting criteria and a planning framework, RE-AIM, to obtain more detail about implementation strategies and desired outcomes. We found that, on average, the three pharmacogenomics implementation projects used more strategies than the disease-focused projects. Overall, projects had four implementation strategies in common; however, operationalization of each differed in accordance with each study’s implementation outcomes. These four common strategies may be important for precision medicine program implementation, and pharmacogenomics may require more integration into clinical care. Understanding how and why these strategies were successfully employed could be useful for others implementing genomic or precision medicine programs in different contexts.

## 1. Introduction

Precision medicine represents a new, emerging paradigm for healthcare by tailoring treatments to individuals on the basis of characteristics that include biological, behavioral, and demographic data. The emergence of precision medicine as a viable approach to healthcare, compared to the traditional one-size-fits-all approach, follows in large part technological advances, such as sequencing the human genome and harnessing big datasets. Because of the size, complexity, and novelty of information needed to practice precision medicine, implementation must include tools to help clinicians and patients interpret and act on the information [1]. These tools include clinical decision supports (CDS), i.e., “guidelines, prompts, and assists” that deliver information at the point of healthcare delivery [2] (p. 2). Typically, CDSs are integrated with the electronic health record (EHR) to provide just-in-time prompts for clinicians or information for patients [3]. In particular, they have proven efficacious for translating genomic medicine into clinical care [4,5].

However, there is little understanding about how to implement interventions that include CDS for interpreting and using genomic information. Although genomic discoveries have exponentially advanced following the Human Genome Project to sequence the complete human genome over 10 years ago, to date, little research has focused on best practices to translate discoveries into routine care [6,7]. Barriers to translation center on a lack of coordinated and systematic processes to educate stakeholders about genomic medicine innovations and challenges in their integration with existing platforms [8]. Implementation science, the scientific study of methods to promote uptake of innovations in real-world settings, can provide guidance on selecting strategies for translating genomic medicine innovations into clinical practice [9]. Unlike quality improvement, which focuses on specific problems within specific settings, implementation science aims to produce generalizable knowledge about ways to improve healthcare delivery. As such, implementation research starts with an underutilized evidence-based practice and focuses on processes to deliver the practice, providing a frame for defining, measuring, and reproducing strategies to improve use of the clinical practice—the “how”—in different contexts [10,11]. 

To better understand processes used to implement genomic medicine-focused CDS, we conducted an in-depth evaluation of implementation strategies across a network focused on implementing genomic medicine, called Implementing Genomics In Practice (IGNITE). Each implementation included a CDS intervention to prompt and support providers to consider genomic information in clinical care [12]. We used implementation science to better understand implementation processes, as well as to identify and describe the common implementation strategies related to each project’s context and implementation outcomes. The approach and results of this work offer an implementation sciences-based frame for guiding and evaluating clinical implementations of genomic interventions. 

## 2. Materials and Methods

### 2.1. Settings

The IGNITE network consisted of six diverse genomic medicine demonstration projects led by academic medical centers allied with community healthcare systems that varied in their goals and approach. Previous publications have described the projects in detail [12,13,14]. In short, three projects implemented different types of pharmacogenomics (PGx) CDS interventions in the EHR (INGENIOUS: Indiana Genomics Implementation, an Opportunity for the Underserved, Indiana University; Genomic Medicine Implementation: the Personalized Medicine Program (PMP), University of Florida; Integrated, Individualized, and Intelligent Prescribing (I^3^P) Network, Vanderbilt University). Three projects had disease focus (PDMP: the Personalized Diabetes Medicine Program at the University of Maryland School of Medicine to identify individuals with monogenic subtypes of common disease; the GUARDD Study: Genetic Testing to Understand and Address Renal Disease Disparities, Icahn School of Medicine at Mount Sinai, to proactively identify patients at risk for chronic disease; Implementation, Adoption, and Utility of Family History in Diverse Care Settings, Duke University to implement a patient-facing web-based family health history-based risk assessment tool integrated with the EHR). All projects implemented CDS tools into Epic EHR, with two projects, INGENIOUS and I^3^P, additionally including homegrown EHRs in some affiliated health systems.

### 2.2. Frameworks

Theoretical frameworks in implementation science offer common terms and definitions to identify and explain complex phenomena experienced across diverse contexts [15]. One highly cited implementation science framework, Reach, Effectiveness, Adoption, Implementation, and Maintenance (RE-AIM), offers dimensions for explicitly reporting key aspects of translating evidence-based interventions into diverse settings, including Reach (R)—the number, proportion, or representativeness of individuals willing to participate and Effectiveness (E)—the impact on outcomes including potential negative effects of the intervention [16,17]. For example, Wu and colleagues (2019) used RE-AIM to illustrate that diverse healthcare settings could successfully implement a new computerized family health history screening tool, although odds of completing the screening decreased with male sex and minority race [18]. Proctor and colleagues (2013) additionally published guidance for reporting implementation strategies, recommending that authors describe specific dimensions such as the stakeholders involved (actors) or when they used the strategy (action), with an eye toward measurement and reproducibility [19]. Powell and colleagues (2015) further developed a taxonomy of evidence-based implementation strategies, known as the Expert Recommendations for Implementing Change (ERIC) project. This taxonomy includes 73 implementation strategies, organized according to nine domains. Prior work has identified ERIC implementation strategies used to meet common barriers for genomic medicine implementation [20]. While the ERIC provides a useful compendium, the full list of strategies, originally developed within the context of mental health research and practice, have yet to be evaluated in conjunction with Proctor’s detailed reporting criteria in the context of genomic medicine implementation.

### 2.3. Procedures

Three implementation scientists on the research team worked with project teams to systematically elicit information about implementation strategies and outcomes in two phases. The IRB approved study procedures.

During the first phase, they developed a web-based, self-administered 15 min structured survey to gather information on implementation strategies used at sites. The format was based on a previously published survey of implementation strategies, in which questions about strategies were organized by nine domains, or clusters, grouping conceptually related strategies together (e.g., using evaluative and iterative strategies) [21,22]. The implementation strategies came from the ERIC, a taxonomy of evidence-based implementation strategies. This taxonomy includes 73 implementation strategies, organized according to nine domains. This survey of IGNITE projects queried the use of 72/73 ERIC implementation strategies, excluding a question about the strategy of “developing academic partnerships”, because it was integral to the consortium as a whole. The survey asked about use of a cluster of strategies generally (e.g., During IGNITE I, did your project use any of these evaluative and iterative strategies to implement your innovation at any of your project sites?) and then specific strategies within each cluster, with the response options of yes, no, and not sure (see Supplementary Materials Files S1 for survey questions). The survey was programmed in Qualtrics (Provo, UT, USA), and a link was emailed to project coordinators at each site for completion. Survey results were analyzed using Microsoft Excel (Redmond, WA, USA).

Common strategies employed by all six projects were identified for follow-up in a second phase. The implementation scientists conducted 30–45 min phone-based qualitative interviews with project coordinators and one principal investigator, with the exception of two projects, in which the principal investigator (PI) responded directly by email. These interviews included questions about implementation outcomes as specified by the RE-AIM planning framework and detailed information about how strategies were deployed, as specified by the “reporting dimensions of implementation strategies” guidance (actor, temporality, action, justification, target) [16,19]. The respondents received the list of questions approximately 1 week before the phone interview and had the opportunity to add more information later by email and telephone (Supplementary Materials Files S1). NVivo 12 software (Melbourne, Australia) was used to manage, organize, and query the qualitative data for analysis.

## 3. Results

### 3.1. Variety of Implementation Strategies Used across the Network

**Figure 1:**
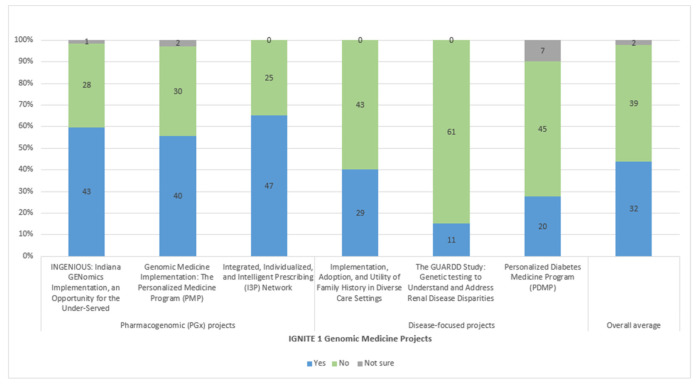
Number of ERIC implementation strategies used by IGNITE implementation project.

On average, IGNITE projects implemented 32 ERIC strategies. The number of strategies used by each project varied, ranging from 11–47 (Figure 1). Each of the three PGx projects used over 40 strategies, while the three disease-focused projects used 11–29 strategies (see Supplementary Materials Files S2 for results).

### 3.2. Common Implementation Strategies Found among Diverse Implementation Projects

**Figure 2:**
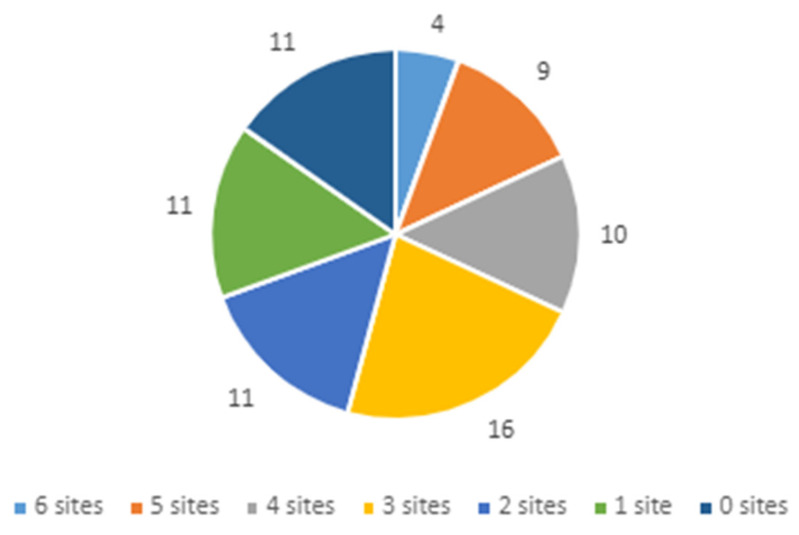
Number of projects implementing each of the 72 ERIC strategies.

Despite the diversity of project goals and approaches, four strategies from three clusters were used across all six projects (Figure 2): (1) developing strategies to obtain and use stakeholder feedback (cluster—using evaluative and iterative strategies), (2) identifying early adopters (cluster—developing stakeholder interrelationships), (3) conducting educational meetings (cluster—training and educating stakeholders), and (4) having an expert meet with clinicians to educate them (cluster—training and educating stakeholders). 

#### 3.2.1. Implementation Strategy 1: Obtaining and Using Stakeholder Feedback (e.g., from Patients, Families, or Providers) to Evaluate and Iteratively Develop the Genomic Program

All projects reported obtaining some form of feedback from stakeholders (Table 1). In all cases, experts were involved; however, the sources of feedback varied (researchers, administrators, community advisory board, clinicians, and patients). Generally, the projects obtained stakeholder feedback before project start and continued throughout, although not necessarily systematically, with the exception of GUARDD, which organized standing Stakeholder Advisory Board meetings. Actions for obtaining stakeholder feedback included a mix of informal and formal steps. For example, Implementation, Adoption, and Utility of Family History in Diverse Care Settings conducted pre-implementation meetings with all clinics and formal assessment with providers throughout, and GUARDD had meetings with their Stakeholder Advisory Board, while others informally asked for feedback during existing meetings with providers. All projects justified using stakeholder feedback to make sure that the project would work at the implementing site, mostly a function of the PI’s prior experience with multi-site and community-based research, such as knowing with whom to engage to ensure buy-in for testing the project. The targets of change from obtaining feedback varied, including understanding leverage points for implementing genomic medicine within healthcare systems, seeking ways to bolster recruitment and retention, or improving provider knowledge about genomic data.

#### 3.2.2. Implementation Strategy 2: Identifying Early Adopters to Develop Stakeholder Interrelationships to Deliver the Genomic Program

All projects, prior to implementation, identified champions, i.e., individuals committed to supporting and promoting implementation of the practice, to help obtain buy-in and enroll participants (Table 2) [23]. Typically, projects did not employ specific, prescribed steps to identify champions, with the exception of Implementation, Adoption, and Utility of Family History in Diverse Care Settings, in which the local PIs were each asked to identify a champion at their place. Otherwise, champions spread the word through educational meetings or helped to inform the projects by working with the PI. Each project largely had a pragmatic reason for using this implementation strategy in that site champions would bring attention to the project among providers or offer access to others for support. This implementation strategy mostly targeted provider knowledge and skill to, in turn, change clinical processes to include the genomic information.

#### 3.2.3. Implementation Strategies 3 and 4: Conducting Educational Meetings and Having an Expert Meet with Clinicians to Train or Educate Providers to Deliver the Genomic Program

We present the two strategies having to do with the training and educating stakeholders strategy cluster together in one table (Table 3), because, although they are discrete strategies according to the ERIC typology, results indicated that they went hand-in-hand for these genomic medicine implementations. Strategies used for “training and educating stakeholders” involved PIs presenting information about their project and protocol along with subject experts to clinicians who would likely be involved with implementation. The Implementation, Adoption, and Utility of Family History in Diverse Care Settings approach differed slightly in that the project crossed clinical areas and the PI had expertise in use of the web-based family health history tool. Projects generally used these strategies during pre-implementation, with the exception of PMP, which used it throughout the study on an ad hoc basis. Research teams did not report formal steps for employing this strategy, with meetings set as needed to educate clinicians or, in the case of the GUARDD study, integrated with regular, standing provider meetings. Generally, projects used these stratagies for pragmatic reasons to make sure that clinicians who would be integral to trial implementation understood and accepted the innovations, protocols, and evidence. Experts were used to engage directly with clinicians by providing first-hand experiences (PMP) and to educate peers about empirical evidence behind the project. Across the board, the action, or target, was to change provider knowledge about the content area and bring them into the fold with study protocol.

### 3.3. Implementation Outcomes

All projects focused on patient-level outcomes to evaluate implementation. Table 4 describes outcomes according to RE-AIM dimensions of Reach, Adoption, and Effectiveness. 

## 4. Discussion

Although we identified common implementation strategies, the detailed reporting criteria revealed different manifestations of the strategies across the projects. For example, all projects employed a strategy for “obtaining and using stakeholder feedback”; however, each project described how they uniquely employed this strategy, including using pre-implementation meetings with clinicians, a stakeholder advisory board reflecting the clinician and patient population [25], involvement of a Clinical and Translational Science Institute, inclusion of patients, and weekly meetings with a multidisciplinary team. Additionally, the strategy for “identifying early adopters” differed across projects, for example, with the Implementation, Adoption, and Utility of Family History in Diverse Care Settings identifying site champions at each clinic who would implement the project versus GUARDD using the project team as champions to increase awareness among providers that they would enroll patients into the study, test them, and return genetic test results. Education strategies varied as well, with, for example, PMP using pharmacists to educate providers via case studies and INGENIOUS training clinicians as part of the study team. This variability in the use of common implementation strategies makes sense when considered alongside each project’s RE-AIM implementation outcomes; each project had a different target for adoption (e.g., four major, diverse healthcare systems in the US versus 15 neighborhood-based clinics in one region) or effectiveness (e.g., feasibility of implementing genetic risk assessment in diverse settings vs. improved individual outcomes through genetic testing). This study underscores the importance of defining mechanisms, i.e., precise descriptions of processes or events through which implementation strategies affect implementation outcomes, in describing and evaluating implementation endeavors in general [26].

A previous analysis identified different strategies used by a number of IGNITE projects to meet specific implementation barriers [8]. These included strategies to integrate genomic data into the EHR and engage participants in genomic medicine projects. The present study adds to that prior work by identifying implementation strategies used as part of overall project plans, rather than a response to specific barriers during the course of implementation. Additionally, the earlier query was conducted while the projects were ongoing, while this one was conducted after external funding ended, allowing project coordinators to reflect on core implementation strategies. Differences could also reflect a need for better refinement of the ERIC typology. Perry et al. (2019) also applied ERIC taxonomy in conjunction with Proctor criteria in the context of cardiac prevention in primary care and suggested revisions to refine the taxonomy, including suggestions to combine strategies just as we did in this report with the education strategies in Table 3 [27]. Despite differences between the two analyses of IGNITE implementation strategies, there was some similarity in a common use of educational strategies to improve clinician knowledge and beliefs. Although the ERIC taxonomy does apply to different health-related areas, further work could focus on developing a version of the ERIC taxonomy specifically for genomic medicine implementation.

The three IGNITE PGx projects each reported using a greater number of implementation strategies than the three disease-focused projects. This difference may reflect more extensive infrastructure used to integrate PGx into routine care, for example, financial billing strategies such as new clinic codes or performance indicators such as turnaround time [28,29,30,31]. Additionally, PGx implementation may require more strategies for training or educating providers on how to interpret and use information for the range of drug-gene pairs included than disease-focused projects [28]. In contrast, the GUARDD project, which relied on the project team rather than providers to return results to patients, used the fewest number of implementation strategies. It could be that this difference between PGx and disease-focused projects dissipates when implementing outside of a funded demonstration project. While this study of the IGNITE consortium focused on common, core strategies, future work could further identify and compare strategies by type of genomic medicine implementation.

This study had several limitations. IGNITE genomic demonstration projects received federal research funding support and, thus, may not represent experiences of those seeking to implement genomic medicine interventions without this kind of support. In addition, reports of strategies used reflect the recall of project coordinators and principal investigators. There might have been other implementation strategies used during the course of project implementation. Additionally, these findings reflect implementation experiences from within US healthcare institutions. As such, there may be different strategies used when initiated by a governmental entity. This kind of approach to specify implementation strategies according to published criteria and definitions could be used to compare implementation by national or regional healthcare systems around the world. However, regardless of these limitations, this paper helps to build the evidence base of strategies for implementing genomic medicine.

## 5. Conclusions

Implementing a genomic medicine service is a daunting task, and this study yields three key lessons to help guide others interested in implementation. Firstly, genomic medicine projects will end up using a variety of strategies tailored to the environment and practice, with the number of strategies among these demonstration projects ranging from 11 to 47. Secondly, the four strategies highlighted in this analysis can serve as a manageable starting point for future implementation. Thirdly, systematic PGx programs, in which patients’ genotypes are made available in the EHR to preemptively guide prescribing, can be complicated to implement; for example, IGNITE PGx projects used more implementation strategies than disease-focused ones. Although this study was not designed to identify which strategies are more critical to implement than others for specific practices or desired endpoints, further work can identify the necessity and sufficiency of particular strategies within specific contexts.

## Figures and Tables

**Table 1:** Specification of “obtaining and using stakeholder feedback” implementation strategy by IGNITE 1 genomic medicine implementation projects.

Project	Actor	Temporality	Action	Justification	Target
	Who were the people actively involved and what were their roles?	What can you tell us about when the strategy was used?	What were the steps in using this strategy?	Please briefly describe the rationale for using it.	What were you trying to change? Were there multiple targets you were trying to change?
INGENIOUS: Indiana Genomics Implementation, an Opportunity for the Underserved	Pharmacogenomics experts on the IGNITE team, lab experts, and providers involved with the projects provided feedback on validation of testing, returning results to providers, and clinical actions	Implementation	During team meetings, obtained feedback from lab experts	To unite key stakeholders	Implement an approach to use pharmacogenomics to guide 27 drug therapies
Genomic Medicine Implementation: The Personalized Medicine Program (PMP)	Principal investigators, project coordinator, and pharmacists sought feedback from providers/directors of Clinical Translational Science Instiute (CTSI) and pathology lab gave feedback	Pre-implementation and implementation	Informally asked for feedback during regular meetings with directors	To engage the appropriate stakeholder groups to ensure program success	Improve feasibility of ordering genetic test and patient clinical outcomes
Integrated, Individualized, and Intelligent Prescribing (I^3^P) Network	Lab operations, health IT, PGx experts, and clinical champions provided feedback on logistics on implementation	Pre-implementation and implementation	Discussed during regularly scheduled meetings	Pragmatic reason/prior experience	Implement an approach to use PGx in diverse clinical settings
Implementation, Adoption, and Utility of Family History in Diverse Care Settings	Genomics Expert Executive Board guided activities/study team tested family history program/Spanish speakers tested Spanish version	Pre-implementation and implementation	Met with clinics to assess implementation readiness and evaluated progress	To ensure the program worked at their site, and to understand how to address clinic barriers	Understand how to incorporate and adapt family history tool in healthcare systems
The GUARDD Study: Genetic testing to Understand and Address Renal Disease Disparities	Principal investigator reviewed study tools (recruitment scripts, informed consent) with stakeholder board	Implementation	Met with Stakeholder Board	To improve program success through using influence from similar target populations	Assess recruitment feasibility
Personalized Diabetes Medicine Program (PDMP)	Principal investigators obtained feedback from clinicans and patients	Pre-implementation and implementation	Included clinical champions in study; interacted with providers at staff meetings, in clinic, by email; informally asked patients in study and from advocacy groups	To obtain feedback to develop study protocol	Provider knowledge about candidates for genetic testing

**Table 2:** Specification of “identifying early adopters” implementation strategy by IGNITE 1 genomic medicine implementation projects.

Project	Actor	Temporality	Action	Justification	Target
	Who were the people actively involved and what were their roles?	What can you tell us about when the strategy was used?	What were steps in using this strategy?	Please briefly describe the rationale for using it.	What were you trying to change? Were there multiple targets you were trying to change?
INGENIOUS: Indiana Genomics Implementation, an Opportunity for the Underserved	Fellows analyzed data/clinicians supervised the fellows	Implementation	Analyzed genetic results, generated recommendations, sent reports to patients’ providers via the Electronic Health Record	To rely on clinicians familiar with recommendations and to have the ability to send EHR notes to providers	Policy change (e.g., generate evidence to get tests reimbursed and clincians to accept it) for 27 different drugs
Genomic Medicine Implementation: The Personalized Medicine Program (PMP)	Clinical champion with relevant experience (in chronic pain) helped implement project at multiple clinics by getting buy-in from medical directors, patients, and providers	Pre-implementation/implementation	Educated providers via lunch and learns, met with medical directors to discuss the project, enrolled patients, wrote study protocol	To use the site champion’s experience in chronic pain to get buy-in and program support from medical directors	Provider knowledge about and skill with using pharmacogenomics and using CYP2D6 information in their prescribing
Integrated, Individualized, and Intelligent Prescribing (I^3^P) Network	Clinical champions on the study team obtained buy-in from clinics to recruit patients	Pre-implementation/implementation	Clinical champions helped to educate providers and encourage adoption	Pragmatic and prior experience	Existing clinical processes
Implementation, Adoption, and Utility of Family History in Diverse Care Settings	Champions from each site enrolled patients and providers and solved issues in clinic	Pre-implementation/implementation	PI identified site champions who were then trained on the project	To allow site champions to highlight importance of program to other providers	Existing clinical processes
The GUARDD Study: Genetic testing to Understand and Address Renal Disease Disparities	Clinical champions on research team obtained buy-in from clinics to recruit patients	Pre-implementation	Clinical site champions presented to providers and answered questions (sometimes these providers became champions)	To make providers aware of the program and how to use program information in their practice	Provider knowledge about genetic testing and acceptability of study
Personalized Diabetes Medicine Program (PDMP)	Principal investigator and fellow championed the project in endocrinology clinics	Pre-implementation	Supported project with initial funding to develop various aspects of the projects, including the genetic test, and to educate providers to incorporate into clinic	To leverage the co-PI’s connections in the field and the fellow’s clinical expertise when incorporating the study into the clinic	Effective implementation by helping to develop logistics

**Table 3:** Specification of “conducting educational meetings” and “having an expert meet with clinicians” implementation strategies by IGNITE 1 genomic medicine implementation projects.

Project	Actor	Temporality	Action	Justification	Target
	Who were the people actively involved and what were their roles?	What can you tell us about when the strategy was used?	What were steps in using this strategy?	Please briefly describe the rationale for using it.	What were you trying to change? Were there multiple targets you were trying to change?
INGENIOUS: Indiana Genomics Implementation, an Opportunity for the Underserved	Pharmacogenmics experts provided training to project clinicians who helped evaluate and return pharmacognomics results and recommendations to participants’ providers	Early implementation	Described project process at meetings/ involved clinicians in project design	To train the project clinicians to in turn consult with providers	Train multiple clinicians to make clinical recommendations
Genomic Medicine Implementation: The Personalized Medicine Program (PMP)	Principal investigator and fellow presented project to providers /pharmacist presented case studies to providers	Project presentation pre-implementation and case studies throughout.	Principal investigator and fellow tailored presentation based on medical director’s knowledge of their patients/pharmacist arranged meeting with providers to present case studies	To ensure providers were engaged and understood the program and how to integrate into workflow	Provider knowledge
Integrated, Individualized, and Intelligent Prescribing (I^3^P) Network	Clinical champions and subject matter experts presented to providers	Early implementation	Presented at exisiting meetings like morning report	Pragmatic	Provider knowledge of study and implementation of PGx testing to encourage buy-in
Implementation, Adoption, and Utility of Family History in Diverse Care Settings	Principal investigator, site champions, and project managers visited each site to discuss project and created training videos and informational packets for providers	Pre- and early implementation	Visited each site to provide them with base study protocol, although sites could revise as needed, and provided ongoing educational sessions as needed	To inform sites about the program and allow sites to adjust protocols as needed	Adapt protocol to fit each site/ provider knowledge about how to implement family history assessment
The GUARDD Study: Genetic testing to Understand and Address Renal Disease Disparities	Primary care providers (from the research team and sites) ran educational sessions with providers/specialists with relevant experience (nephrologists, geneticists) developed educational materials/genetic counselor trained site coordinator to return results	Pre-implementation	Asked for time at existing meetings	To ensure providers were trained and received training from peers with similar training backgrounds	Provider understanding of project and expectations
Personalized Diabetes Medicine Program (PDMP)	Principal investigator and study geneticists promoted the project among clinicians/external speakers provided seminars to clinicians	Early implementation	Conducted educational sessions as needed and expert seminars about project intermittently	To make sure providers understood the project, get their buy-in	Maximize provider uptake by increasing knowledge about monogenic types of diabetes, improving case identification, and in turn increasing clinic referrals

**Table 4:** Implementation outcomes and strategies of IGNITE 1 genomic medicine project.

Genomic Medicine Project	Implementation Outcomes			Implementaiton Strategies
	Reach ^1^	Adoption ^1^	Effectiveness ^1^	
	Who actually was exposed to the service?/ Who is or was intended to benefit from your genomic service?	Where is or was the program applied and who applied it?	What is or was the most important benefit you are or were trying to achieve? Were there negative outcomes?	Summary and interpretation
INGENIOUS: Indiana Genomics Implementation, an Opportunity for the Underserved	1309/4380 patients newly prescribed one of 27 different drugs that have clinically actionable genetic variants associated with them; approximately 20% of patients would carry an actionable genetic variant and benefit from a change in their therapy	Indiana University Health (state-wide) and Eskenazi Health (county hospital) healthcare systems, delivered by mostly MDs of multiple disciplines	Improved efficacy and reduced side-effects of the drug therapies; no negative outcomes	Pharmacogenomics experts trained study team clinicians to make recommendations through the EHR to providers who had prescribed one of the drugs with actionable variants
Genomic Medicine Implementation: The Personalized Medicine Program (PMP)	>5000 patients from diverse backgrounds and settings/NA ^2^	Implemented pharmacogenetic testing into clinical practice in 3 hospitals, 23 different clinics, including in academic medical centers, and primary and specialty care settings for 12 different patient populations	Use of genetic testing for drug prescribing (e.g., reduced cardiovascular adverse events); no negative outcomes	Employed relevant multidisciplinary expertise (e.g., clinical champions, pharmacogenomics, pathology, translational medicine) to not only develop project but also engage and educate primary care providers
Integrated, Individualized, and Intelligent Prescribing (I^3^P) Network	25,777 across four diverse healthcare systems in Tennessee, North and South Dakota, and Wisconsin/NA ^2^	VUMC, Advocate Aurora Health, Meharry Medical College, Sanford Health	Uptake in PGx testing and change in treatment; PGx recommendations were followed 50% to 80% of the time; one planned site could not implement PGx testing due to policy issues	Feedback on logistics from diverse stakeholders helped to plan around unique policy and implementation issues at sites, with many difficult to anticipate; providers were receptive to education and PGx recommendations
Implementation, Adoption, and Utility of Family History in Diverse Care Settings	2514/172,160 primary care patients across 5 health systems	28 primary care practices across 4 major healthcare delivery systems in the United States, generally delivered by primary care providers and other healthcare providers, such as nurses, as desired by sites	Feasiblity of implementing genetic risk testing across diverse settings; two negative outcomes: one site dropped out because of feasiblity issues and one clinic dropped out because of issues with time commitment	Conducted pre-implementation site assessments to support local adaptations across the 5 diverse healthcare systems while maintaining project fidelity
The GUARDD Study: Genetic testing to Understand and Address Renal Disease Disparities	2052/7959 eligible adults identified through the EHR ^3^	15 primary care sites, some part of a large academic institution and others from a network of federal qualified health centers in different NYC neighborhoods, delivered by research team	Systolic blood pressure (SBP) decrease: greatest in APOL1-positive compared to APOL1-negative and control groups at 3 months; improved patient health outcomes through genetic testing and information provided to patients and and their providers	Used participatory research approach to inform study tools and educate providers about genetic risk/testing at all participating clinical sites
Personalized Diabetes Medicine Program	2522 patients with diabetes or prediabetes screened in diabetes clinic waiting rooms or patient portal, referred by providers, or referred by patients themselves/NA ^2^	4 endocrinology clinics across 4 healthcare delivery systems, delivered by research team	Improve the identification and diagnosis of patients with monogenic diabetes to enable individualized treatment	Engaged experts in the specific therapeutic area, monogenic diabetes, as part of the study team, to develop protocol and educate providers on how to identify patients with the screening tool

^1^ Glasgow, R.E., et al., RE-AIM Planning and Evaluation Framework: Adapting to New Science and Practice With a 20-Year Review. Frontiers in Public Health, 2019. **7**(64); ^2^ NA = data not available; ^3^ Horowitz, C.R.; Sabin, T.; Ramos, M.; Richardson, L.D.; Hauser, D.; Robinson, M.; Fei, K. Successful recruitment and retention of diverse participants in a genomics clinical trial: A good invitation to a great party. Genet Med. **2019**, *21*, 2364–2370. Epub 2019/04/06. doi:10.1038/s41436-019-0498-x. PubMed PMID: 30948857 [24].

## Data Availability

The data presented in this study are available in Supplementary Materials Files S2.

## Supplementary Materials

The following are available online at https://www.mdpi.com/article/10.3390/jpm11070647/s1, Supplementary Materials Files S1: Survey and Interview Questions, Supplementary Materials Files S2: Survey Responses.
