# Supplementary material for: Strategies to Integrate Genomic Medicine into Clinical Care: Evidence from the IGNITE Network
Source: J Pers Med. 2021 Jul 8;11(7):647. doi: 10.3390/jpm11070647 (PMC8306482; doi:10.3390/jpm11070647)
Supplement: Supplementary file 1 [file jpm-11-00647-s001.zip › jpm-1267590-supplementary.pdf]

Supplementary Materials Files 1: Survey and Interview Questions

Survey Questions (phase 1)

Q1.1 The IGNITE I Common Measures Group would like to learn about implementation strategies used over the course of your IGNITE pilot project.

This information will be aggregated with others' for **a cross-network paper** that describes aspects of common implementation strategies to guide future implementation of genomic medicine.

You are not required to take part in this survey. Your participation is entirely voluntary. You can refuse to participate now or you can withdraw at any time after giving your consent. We will also be conducting a telephone interview and will reach out to ask for your participation after the survey process is completed. Survey completion is not required for the telephone interview.

There is minimal risk associated with this study. While we make every effort to maintain the confidentiality of responses, it is possible for confidentiality to be lost. Procedures are in place to protect participants' privacy. Study data are immediately uploaded to a study specific folder in Duke Box and not saved on an individual computer. If results of this study are reported in medical journals or at meetings, you will not be identified by name, by recognizable photograph, or by any other means without your specific consent. Despite these precautions a loss of confidentiality may occur.

You may not personally be helped by being in this study, but your participation may lead to knowledge that will help others.

If you consent, please continue.

This should take about **15 minutes** to complete.

If you have questions, please contact Nina Sperber at nina.sperber@duke.edu.

Thank you for your participation.

Q2.1 **During IGNITE I, did your project use any of these evaluative and iterative strategies to implement your innovation at any of your project sites?**

For each of the implementation strategies, please select “yes” if your project has used the strategy, “no” if it has not, or “not sure” if you do not know.

	Yes (1)	No (2)	Not sure (3)
1. Assess for readiness and identify barriers and facilitators to change (e.g., administer the organizational readiness to change survey) (1)	○	○	○
2. Develop strategies to obtain and use stakeholder feedback (e.g., from patients, families, or providers) (2)	○	○	○
3. Intentionally examine efforts to implement the genomic service (3)	○	○	○
4. Develop and implement tools that monitor clinical processes and/or outcomes for the purpose of quality assurance and improvement (4)	○	○	○
5. Develop and organize systems that monitor clinical processes and/or outcomes for the purpose of quality assurance and improvement (5)	○	○	○
6. Develop a formal implementation blueprint (6)	○	○	○
7. Conduct local needs assessment (i.e., collect data to determine how to best change things) (7)	○	○	○
8. Stage implementation scale up (8)	○	○	○
9. Obtain and use patient/consumer and family feedback (9)	○	○	○

10. Conduct cyclical small tests of change (10)	○	○	○

Q3.1 **During IGNITE I, did your project employ any of these activities to provide interactive assistance to promote your genomic service at any of your project sites?**

For each of the implementation strategies, please select “yes” if your project has used the strategy, “no” if it has not, or “not sure” if you do not know.

	Yes (1)	No (2)	Not sure (3)
11. Use outside assistance often called "facilitation" (e.g., coaching, education, and/or feedback from the facilitator) (1)	○	○	○
12. Have someone from the project site (often called "local technical assistance") tasked with assisting the site (2)	○	○	○
13. Provide clinical supervision (e.g., train providers) (3)	○	○	○
14. Use a centralized system (i.e., from the university) to deliver facilitation (4)	○	○	○

Q4.1 **During IGNITE I, did your project employ any of these activities to tailor the genomic service to your different sites?**

For each of the implementation strategies, please select “yes” if your project has used the strategy, “no” if it has not, or “not sure” if you do not know.

	Yes (1)	No (2)	Not sure (3)
15. Tailor strategies to deliver genomic services (i.e., alter genomic service delivery to address barriers that you identified in your population using data you collected) (1)	○	○	○
16. Promote adaptability (i.e., identify the ways that genomic services can be tailored to meet local needs and clarify which elements of care must be maintained to preserve fidelity) (2)	○	○	○
17. Use data experts to manage genomic services (3)	○	○	○
18. Use data warehousing techniques (4)	○	○	○

Q5.1 **During IGNITE I, did your project employ any of these activities to develop stakeholder interrelationships to deliver genomic services at your sites?**

For each of the implementation strategies, please select “yes” if your project has used the strategy, “no” if it has not, or “not sure” if you do not know.

	Yes (1)	No (2)	Not sure (3)
19. Identify and prepare champions (i.e., select key individuals who will dedicate themselves to delivering genomic services) (1)	○	○	○
20. Organize clinician implementation team meetings (e.g., support teams of clinicians who are delivering genomic services with time to share the lessons learned and support one another's learning) (2)	○	○	○
21. Recruit, design, and/or train leaders (3)	○	○	○
22. Inform local opinion leaders about advances in genomic services (4)	○	○	○
23. Build a local coalition/team to address challenges (5)	○	○	○
24. Obtain formal written commitments from key partners that state what they will do to deliver genomic services (6)	○	○	○
25. Identify early adopters (7)	○	○	○
26. Conduct local consensus discussions (i.e., determine how to change things by having meetings with local leaders and providers) (8)	○	○	○

27. Share the knowledge gained from quality improvement efforts with other sites outside of your project (9)	○	○	○
28. Use advisory boards and interdisciplinary workgroups to provide input into genomic service policies and elicit recommendations (10)	○	○	○
29. Seek the guidance of experts in implementation (11)	○	○	○
30. Use modeling or simulated change (12)	○	○	○
31. Visit other sites outside your medical center to try to learn from their experiences (13)	○	○	○
32. Involve executive boards (14)	○	○	○
33. Develop an implementation glossary (i.e., a list of terms describing your genomic service and stakeholders) (15)	○	○	○
34. Build on existing high-quality working relationships and networks to promote information sharing and problem solving related to delivering genomic services (16)	○	○	○

Q6.1 **During IGNITE I, did your project employ any of these activities to train or educate providers to deliver genomic services at your site?**

For each of the implementation strategies, please select “yes” if your project has used the strategy, “no” if it has not, or “not sure” if you do not know.

	Yes (1)	No (2)	Not sure (3)
35. Provide ongoing genomics training (1)	◯	◯	◯
36. Provide ongoing consultation with one or more genomics experts (2)	◯	◯	◯
37. Develop formal educational materials (3)	◯	◯	◯
38. Make training dynamic (e.g., vary the information delivery methods to cater to different learning styles when presenting new information) (4)	◯	◯	◯
39. Distribute educational materials (e.g., guidelines, manuals or toolkits) (5)	◯	◯	◯
40. Train designated clinicians to train others (e.g., guidelines, manuals or toolkits) (6)	◯	◯	◯
41. Conduct educational meetings (7)	◯	◯	◯
42. Have an expert meet with clinicians to educate them (8)	◯	◯	◯
43. Facilitate the formation of groups of providers and foster a collaborative learning environment (9)	◯	◯	◯
44. Give providers opportunities to shadow other experts in genomics (10)	◯	◯	◯
45. Use educational institutions to train clinicians (11)	◯	◯	◯

Q7.1 **During IGNITE I, did your project use any of these activities to support clinicians in implementing genomic services?**

For each of the implementation strategies, please select “yes” if your project has used the strategy, “no” if it has not, or “not sure” if you do not know.

	Yes (1)	No (2)	Not sure (3)
46. Facilitate relay of clinical data to providers (1)	○	○	○
47. Remind clinicians (2)	○	○	○
48. Develop resource sharing agreements (3)	○	○	○
49. Revise professional roles (4)	○	○	○
50. Create new clinical teams (5)	○	○	○

Q8.1 **During IGNITE I, did your project use any of these activities to engage consumers?**

For each of the implementation strategies, please select “yes” if your project has used the strategy, “no” if it has not, or “not sure” if you do not know.

	Yes (1)	No (2)	Not sure (3)
51. Involve patients/consumers and family members (1)	○	○	○
52. Intervene with patients/consumers to promote uptake and adherence to the genomic service (2)	○	○	○
53. Engage in efforts to prepare patients to be active participants in the genomic service (3)	○	○	○
54. Promote demand for genomic services through any other means (4)	○	○	○
55. Use mass media (e.g., local public service announcements, newsletters, online/social media outlets) to reach large numbers of people (5)	○	○	○

Q9.1 **During IGNITE I, did your project use any of these financial strategies to promote genomic services at your sites?**

For each of the implementation strategies, please select “yes” if your project has used the strategy, “no” if it has not, or “not sure” if you do not know.

	Yes (1)	No (2)	Not sure (3)
56. Respond to proposals to deliver genomic services (1)	○	○	○
57. Access new funding (2)	○	○	○
58. Place genomic services on fee for service lists (3)	○	○	○
59. Alter incentive/allowance structures (4)	○	○	○
60. Change billing (e.g., create new clinic codes for billing for genomic services) (5)	○	○	○
61. Alter patient fees (6)	○	○	○
62. Use other payment schemes (7)	○	○	○
63. Provide financial disincentives for failure to implement or use the clinical innovations (8)	○	○	○
64. Use capitated payments (9)	○	○	○

Q10.1 **During IGNITE I, did your project change infrastructure to implement genomic services in any of the following ways?**

For each of the implementation strategies, please select “yes” if your project has used the strategy, “no” if it has not, or “not sure” if you do not know.

	Yes (1)	No (2)	Not sure (3)
65. Mandate changes (1)	○	○	○
66. Change the record systems (e.g., locally create new or update to existing clinical reminder, develop standardized notes templates) (2)	○	○	○
67. Change physical structure and equipment (3)	○	○	○
68. Create or change credentialing and/or licensure standards (e.g., change of scopes of practice or service agreements) (4)	○	○	○
69. Change the location of clinical service sites (5)	○	○	○
70. Change accreditation or membership requirements (6)	○	○	○
71. Develop a separate organization or group responsible for disseminating genomic services (outside of IGNITE) (7)	○	○	○
72. Participate in liability reform efforts that make clinicians more willing to deliver the clinical innovation (8)	○	○	○

Interview Questions (phase 2)

Implementation Outcomes

Questions in this section are related to the general genomics program at your site.

1. Who was *intended* to benefit from the program? (If possible, please provide approximate percentage and types of patients; if metrics not available, please describe qualitatively)
    a. Description of patients:
    b. Quantitative:
    c. OR Qualitative:

2. Who *actually* used the program? (If possible, please provide approximate percentage and types of patients; if metrics not available, please describe qualitatively)
    a. Description of patients:
    b. Quantitative:
    c. OR Qualitative:

3. a. What is or was the most important improvement the program was trying to achieve?

b. Were there negative outcomes?

4. a. Where is or was the program implemented (e.g., types of clinics, hospital departments, or community-based agencies)?

b. Which type of provider delivered it (e.g., pharmacists, nurses, primary care providers)?

Implementation Strategies

Questions in this section are related to how your site incorporated four specific implementation strategies.

Strategy 1: Obtain and use stakeholder feedback (e.g., from patients, families, or providers) to evaluate and iteratively develop the genomic program

1. Who were actively involved and what were their roles?
2. What can you tell us about when the strategy was used? (e.g., what stage or phase of project)
3. What were steps in using this strategy?
4. Please briefly describe the rationale for using it. (e.g., prior experience, empirical evidence, or pragmatic reason)
5. a. What were you trying to change? (e.g., policy context, provider knowledge or skill)

b. Were there multiple targets you were trying to change?

Strategy 2: Identify early adopters to develop stakeholder interrelationships to deliver the genomic program

1. Who were actively involved and what were their roles?
2. What can you tell us about when the strategy was used? (e.g., what stage or phase of project)
3. What were steps in using this strategy?
4. Please briefly describe the rationale for using it. (e.g., prior experience, empirical evidence, or pragmatic reason)
5. a. What were you trying to change? (e.g., policy context, provider knowledge or skill)

b. Were there multiple targets you were trying to change?

Strategy 3: Conduct educational meetings to train or educate providers to deliver the genomic program

1. Who were actively involved and what were their roles?
2. What can you tell us about when the strategy was used? (e.g., what stage or phase of project)
3. What were steps in using this strategy?
4. Please briefly describe the rationale for using it. (e.g., prior experience, empirical evidence, or pragmatic reason)
5. a. What were you trying to change? (e.g., policy context, provider knowledge or skill)

b. Were there multiple targets you were trying to change?

Strategy 4: Have an expert meet with clinicians to educate them to deliver the genomic program

1. Who were actively involved and what were their roles?
2. What can you tell us about when the strategy was used? (e.g., what stage or phase of project)
3. What were steps in using this strategy?
4. Please briefly describe the rationale for using it. (e.g., prior experience, empirical evidence, or pragmatic reason)
5. a. What were you trying to change? (e.g., policy context, provider knowledge or skill)

b. Were there multiple targets you were trying to change?

Supplementary Materials Files 2: Survey Responses

	IGNITE I Site						
Question	Duke University	Indiana University	Icahn School of Medicine at Mount Sinai	University of Florida	University of Maryland	Vanderbilt University	% of sites using strategy
**During IGNITE I, did your project use any of these evaluative and iterative strategies to implement your innovation at any of your project sites?**							
1.Assess for readiness and identify barriers and facilitators to change (e.g., administer the organizational readiness to change survey)	Yes	Yes	No	Yes	Yes	No	67%
2.Develop strategies to obtain and use stakeholder feedback (e.g., from patients, families, or providers)	Yes	Yes	Yes	Yes	Yes	Yes	100%
3.Intentionally examine efforts to implement the genomic service	No	Yes	No	Yes	No	Yes	50%
4.Develop and implement tools that monitor clinical processes and/or outcomes for the purpose of quality assurance and improvement	No	Yes	No	Yes	No	Yes	50%
5.Develop and organize systems that monitor clinical processes and/or outcomes for the purpose of quality assurance and improvement	No	Yes	No	Yes	No	Yes	50%
6.Develop a formal implementation blueprint	Yes	Yes	No	No	Not sure	No	33%
7.Conduct local needs assessment (i.e., collect data to determine how to best change things)	Yes	No	No	Yes	Not sure	Yes	50%
8.Stage implementation scale up	Yes	No	No	Yes	No	Yes	50%
9.Obtain and use patient/consumer and family feedback	Yes	No	No	Yes	Not sure	Yes	50%
10.Conduct cyclical small tests of change	No	No	No	No	Not sure	Yes	17%
**During IGNITE I, did your project employ any of these activities to provide interactive assistance to promote your genomic service at any of your project sites?**							
11.Use outside assistance often called "facilitation" (e.g., coaching, education, and/or feedback from the facilitator)	No	No	No	No	No	No	0%
12. Have someone from the project site (often called "local technical assistance") tasked with assisting the site	Yes	Yes	No	Not sure	Yes	Yes	67%
13.Provide clinical supervision (e.g., train providers)	Yes	Yes	No	Yes	Yes	Yes	83%
14.Use a centralized system (i.e., from the university) to deliver facilitation	Yes	Yes	No	Not sure	No	No	33%
**During IGNITE I, did your project employ any of these activities to tailor the genomic service to your different sites?**							
15.Tailor strategies to deliver genomic services (i.e., alter genomic service delivery to address barriers that you identified in your population using data you collected)	Yes	Yes	No	Yes	Not sure	Yes	67%
16.Promote adaptability (i.e., identify the ways that genomic services can be tailored to meet local needs and clarify which elements of care must be maintained to preserve fidelity)	Yes	Yes	No	Yes	No	Yes	67%
17.Use data experts to manage genomic services	No	Yes	No	Yes	No	Yes	50%
18.Use data warehousing techniques	No	Yes	Yes	No	No	Yes	50%
**During IGNITE I, did your project employ any of these activities to develop stakeholder interrelationships to deliver genomic services at your sites?**							

19. Identify and prepare champions (i.e., select key individuals who will dedicate themselves to delivering genomic services)	Yes	Yes	No	Yes	Yes	Yes	83%
20. Organize clinician implementation team meetings (e.g., support teams of clinicians who are delivering genomic services with time to share the lessons learned and support one another's learning)	No	No	No	Yes	No	Yes	33%
21. Recruit, design, and/or train leaders	Yes	Yes	No	Yes	Yes	Yes	83%
22. Inform local opinion leaders about advances in genomic services	No	Yes	Yes	Yes	No	Yes	67%
23. Build a local coalition/team to address challenges	No	Yes	No	Yes	No	Yes	50%
24. Obtain formal written commitments from key partners that state what they will do to deliver genomic services	Yes	Yes	No	No	No	No	33%
25. Identify early adopters	Yes	Yes	Yes	Yes	Yes	Yes	100%
26. Conduct local consensus discussions (i.e., determine how to change things by having meetings with local leaders and providers)	No	Yes	No	Yes	No	Yes	50%
27. Share the knowledge gained from quality improvement efforts with other sites outside of your project	Yes	Yes	No	Yes	Yes	Yes	83%
28. Use advisory boards and interdisciplinary workgroups to provide input into genomic service policies and elicit recommendations	Yes	No	Yes	Yes	No	Yes	67%
29. Seek the guidance of experts in implementation	Yes	No	Yes	Yes	No	No	50%
30. Use modeling or simulated change	No	Yes	No	No	No	Yes	33%
31. Visit other sites outside your medical center to try to learn from their experiences	No	Yes	No	No	Yes	Yes	50%
32. Involve executive boards	No	Yes	No	Yes	No	Yes	50%
33. Develop an implementation glossary (i.e., a list of terms describing your genomic service and stakeholders)	Yes	Yes	No	No	No	Yes	50%
34. Build on existing high-quality working relationships and networks to promote information sharing and problem solving related to delivering genomic services	Yes	Yes	No	Yes	Not sure	Yes	67%
**During IGNITE I, did your project employ any of these activities to train or educate providers to deliver genomic services at your site?**							
35. Provide ongoing genomics training	No	Yes	No	Yes	Yes	Yes	67%
36. Provide ongoing consultation with one or more genomics experts	No	Yes	No	Yes	No	Yes	50%
37. Develop formal educational materials	Yes	Yes	No	Yes	No	Yes	67%
38. Make training dynamic (e.g., vary the information delivery methods to cater to different learning styles when presenting new information)	No	No	No	Yes	No	No	17%
39. Distribute educational materials (e.g., guidelines, manuals or toolkits)	Yes	Yes	No	Yes	No	Yes	67%
40. Train designated clinicians to train others (e.g., guidelines, manuals or toolkits)	No	Yes	No	Yes	No	No	33%
41. Conduct educational meetings	Yes	Yes	Yes	Yes	Yes	Yes	100%
42. Have an expert meet with clinicians to educate them	Yes	Yes	Yes	Yes	Yes	Yes	100%
43. Facilitate the formation of groups of providers and foster a collaborative	No	Not sure	No	No	No	Yes	17%

44.Give providers opportunities to shadow other experts in genomics	No	No	No	No	No	No	0%
45.Use educational institutions to train clinicians	No	No	No	No	No	No	0%
**During IGNITE I, did your project use any of these activities to support clinicians in implementing genomic services?**							
46.Facilitate relay of clinical data to providers	No	Yes	Yes	Yes	Yes	Yes	83%
47.Remind clinicians	Yes	Yes	No	Yes	Yes	Yes	83%
48.Develop resource sharing agreements	No	Yes	No	No	No	No	17%
49.Revise professional roles	No	No	No	No	No	No	0%
50.Create new clinical teams	No	No	No	No	No	Yes	17%
**During IGNITE I, did your project use any of these activities to engage consumers?**							
51.Involve patients/consumers and family members	No	No	Yes	No	Yes	Yes	50%
52.Intervene with patients/consumers to promote uptake and adherence to the genomic service	No	No	Yes	No	Yes	No	33%
53.Engage in efforts to prepare patients to be active participants in the genomic service	Yes	No	No	No	No	No	17%
54.Promote demand for genomic services through any other means	No	Yes	No	No	Not sure	Yes	33%
55.Use mass media (e.g., local public service announcements, newsletters, online/social media outlets) to reach large numbers of people	No	Yes	No	No	Yes	No	33%
**During IGNITE I, did your project use any of these financial strategies to promote genomic services at your sites?**							
56.Respond to proposals to deliver genomic services	Yes	Yes	No	Yes	Yes	Yes	83%
57.Access new funding	Yes	Yes	No	Yes	Yes	Yes	83%
58. Place genomic services on fee for service lists	No	Yes	No	Yes	No	No	33%
59.Alter incentive/allowance structures	No	No	No	No	No	Yes	17%
60.Change billing (e.g., create new clinic codes for billing for genomic services)	No	Yes	No	Yes	No	No	33%
61.Alter patient fees	No	No	No	No	No	Yes	17%
62.Use other payment schemes	No	No	No	No	No	No	0%
63.Provide financial disincentives for failure to implement or use the clinical innovations	No	No	No	No	No	No	0%
64.Use capitated payments	No	No	No	No	No	No	0%
**During IGNITE I, did your project change infrastructure to implement genomic services in any of the following ways?**							
65.Mandate changes	No	No	No	No	No	No	0%
66.Change the record systems (e.g., locally create new or update to existing clinical reminder, develop standardized notes templates)	Yes	Yes	No	Yes	Yes	Yes	83%
67.Change physical structure and equipment	No	No	No	No	No	Yes	17%
68.Create or change credentialing and/or licensure standards (e.g., change of scopes of practice or service agreements)	No	No	No	Yes	No	No	17%
69.Change the location of clinical service sites	No	No	No	No	No	No	0%
70.Change accreditation or membership requirements	No	No	No	No	No	No	0%
71.Develop a separate organization or group responsible for disseminating genomic services (outside of IGNITE)	No	No	No	No	No	Yes	17%
72.Participate in liability reform efforts that make clinicians more willing to deliver the clinical innovation	No	No	No	No	No	No	0%
